# Neurostimulation in Childhood Epilepsy

**DOI:** 10.1007/s13312-025-00063-z

**Published:** 2025-04-07

**Authors:** Soumya Ghosh, Lakshmi Nagarajan

**Affiliations:** 1 Children’s Neuroscience Service, Dept of Neurology, Perth Children’s Hospital, Nedlands, WA 6009 Australia; 2 Perron Institute for Neurological and Translational Science, University of Western Australia, Nedlands, WA 6009 Australia; 3 School of Medicine, University of Western Australia, Nedlands, WA 6009 Australia

**Keywords:** Deep brain stimulation, Drug resistant epilepsy, Non-invasive brain stimulation, Responsive neurostimulation, Vagus nerve stimulation

## Abstract

Epilepsy is a common and debilitating neurological disorder in children, and approximately a third of them have ongoing seizures despite adequate trial of antiseizure medications. Neurostimulation approaches may be an option for those with drug resistant epilepsy. Several invasive and non-invasive devices have been trialled and found to be effective in reducing seizure burden in drug resistant epilepsy. These techniques appear to be safe and well tolerated. We review the available evidence for the use of these devices, including vagus nerve stimulation, deep brain stimulation, responsive neurostimulation, chronic subthreshold cortical stimulation, transcranial magnetic stimulation and transcranial direct current stimulation. The results of trials are promising but there are fewer studies in children. Apart from vagus nerve stimulation, none of the other neurostimulation techniques are currently approved for use in children and their use is off-label or in clinical trials. Further well-designed trials are needed, especially in children, to identify the most effective neurostimulation options and optimal parameters for improvement of seizure burden and quality of life. Neurostimulation techniques are also being trialled for treatment of refractory status epilepticus, but lack of robust evidence (mainly case studies or case series reports) makes it difficult to predict therapeutic benefit at present.

## Introduction

Epilepsy is a common neurological disorder in children and treatment is mainly based on antiseizure medications. About 30% of children with epilepsy have Drug Resistant Epilepsy (DRE) defined by failure to attain seizure freedom despite adequate trials of antiseizure medications [1]. Children with DRE face serious consequences including interference with schooling, loss of independence, social isolation, risk of injury, depression, cognitive decline, and risk of death. For selected children with focal epilepsy, resective surgery is a good treatment option [2]. Neurostimulation is an option for those who are not eligible for resective surgery, such as those with primary generalized epilepsy, developmental and epileptic encephalopathy, epileptic foci in eloquent cortex or when no localization can be found on multimodal assessment [3, 4]. Neurostimulation techniques are also being trialled for the treatment of refractory status epilepticus but currently there is less evidence (mainly case studies or case series) for their use [5]. Other non-pharmacological therapies used for DRE include ketogenic diet.

**Fig. 1:**
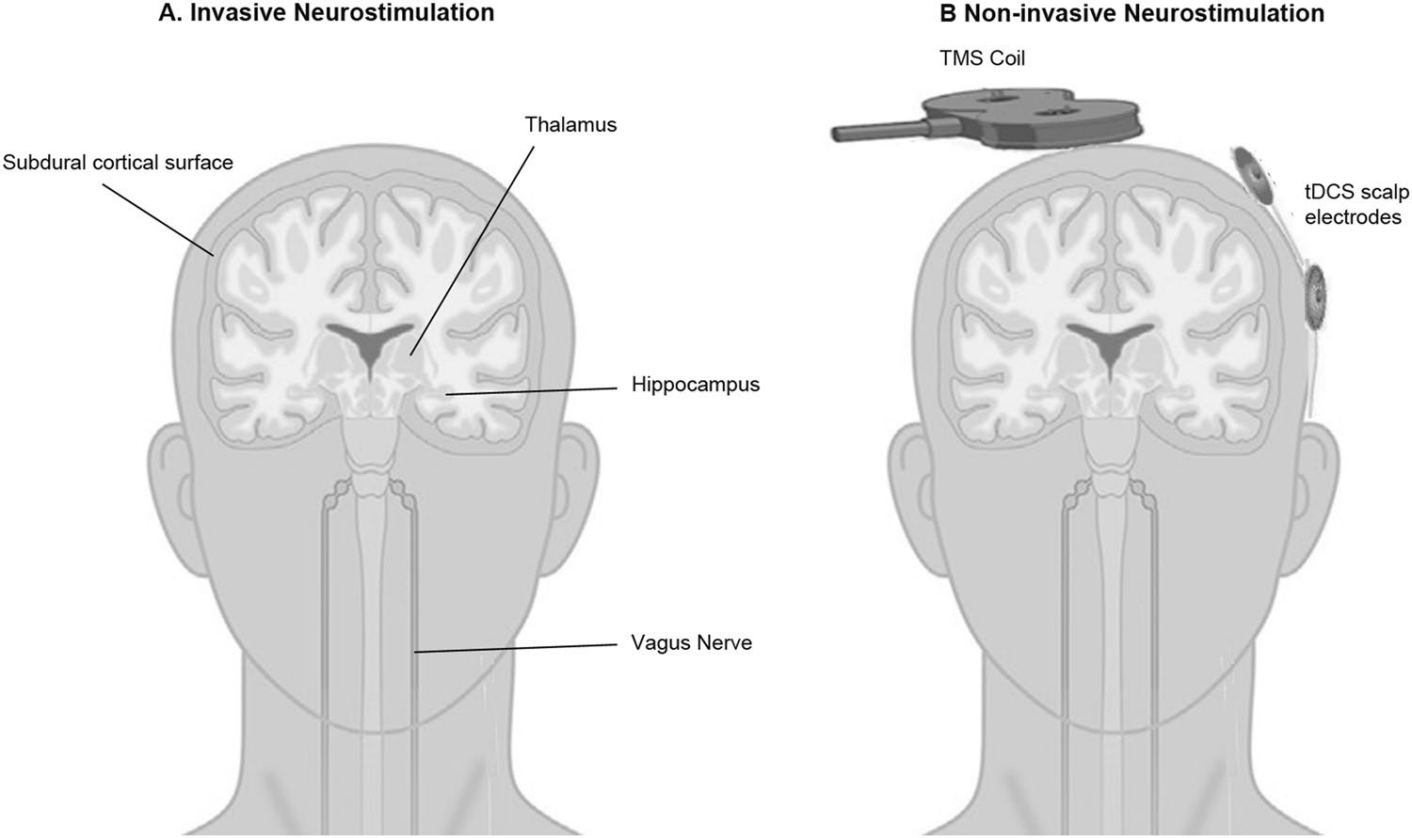
Neurostimulation Techniques. Illustration of common invasive (**A**) and non-invasive (**B**) neurostimulation techniques for treatment of epilepsy. Figure 1A shows the target of invasive stimulation electrodes including the cortical surface (subdural strips or grid for RNS, CSCS), thalamic nuclei (ANT or CNT for DBS), hippocampus and vagus nerve. Figure 1B shows positioning of TMS coil and tDCS scalp electrodes over the region of interest (often the seizure onset zone). ANT, Anterior Nucleus of Thalamus; CNT, Centromedian Nucleus of Thalamus; CSCS, Chronic Subthreshold Cortical Stimulation; RNS, Responsive Neurostimulation; tDCS, transcranial Direct Current Stimulation; TMS, Transcranial Magnetic Stimulation

Neurostimulation for epilepsy involves electrical stimulation of the nervous system with the goal of reducing seizure frequency and severity [3, 4, 6]. There are many invasive and non-invasive neurostimulation devices and modalities currently available, and newer paradigms and techniques are under investigation. Some modalities target the seizure-onset zone for the individual patient, while others target regions connected to and affecting the seizure-related neural networks (Fig. 1). Some devices deliver continuous stimulation (open-loop), whereas others sense brain activity, and provide stimulation based upon detected events (closed-loop). Common invasive neurostimulation devices include vagus nerve stimulation (VNS), deep brain stimulation (DBS), responsive neurostimulation (RNS) and chronic subthreshold cortical stimulation (CSCS) (Table 1, Fig. 1). Non-invasive techniques do not require permanent implantation of a device and stimulation is applied externally and intermittently to the nervous system; commonly used modalities include transcranial magnetic stimulation (TMS) and transcranial electrical stimulation. Most neurostimulation trials and treatments have been conducted in adults with less experience and fewer high-quality evidence in the paediatric age group [7]. Neurostimulation devices, apart from VNS, are not currently approved for use in children and are used off-label or in clinical trials [7]. The cost of neurostimulation devices and implants are high and may not be affordable in many resource-limited settings. These techniques require high levels of expertise for the implantation and programming of the devices, which are available only in select clinical centres. This review provides an overview of the indications, advantages, and limitations of these treatments.

**Table 1:** Summary of common neurostimulation devices used to treat epilepsy

Type of stimulation	Battery location	MRI compatible	Indications	Evidence in Children	Adverse Effects
*Invasive Stimulation*					
Vagus Nerve Stimulation	Chest wall	Yes	Focal and generalized epilepsy	Well studied including 1 RCT	Hoarseness, Cough, Pain, Dyspnea, sleep disorders
Deep Brain Stimulation	Chest wall	Yes	Focal and Generalized epilepsy	Promising results in the short term	Hemorrhage, infection, lead fracture, lead malposition, headache, confusion, dysarthria
Responsive Neurostimulation	Cranium	No	Focal and Generalized epilepsy	Promising results in the short term	Hemorrhage, infection, lead fracture, lead malposition
Chronic Subthreshold Cortical Stimulation	Chest wall	No	Focal epilepsy	Promising results in the short term	Hemorrhage, infection, lead fracture, lead malposition
*Non-invasive Stimulation*					
Transcranial Magnetic Stimulation	N/A	N/A	Focal epilepsy, Status Epilepticus	Promising results in the short term	Rare seizures, headache, dizziness, pain, sleep disturbance
Transcranial Direct Current Stimulation	N/A	N/A	Focal epilepsy, Status Epilepticus	Small RCTs, promising results in the short term	Rare seizures, headache, local itchiness/irritation

MRI, magnetic resonance imaging; N/A, not applicable; RCT, randomised control trial

## Vagus Nerve Stimulation

Vagus nerve stimulation is the best studied neurostimulation modality and has emerged as an important treatment option for children with DRE. It has been found to be effective in focal, generalized and syndromic epilepsy and should be considered for children above the age of 4 years with poorly controlled epilepsy in spite of adequate medical treatment and appropriate evaluation for dietary treatment (e.g. ketogenic diet) and surgical options [3, 4, 8]. VNS has been approved for use in children as young as 4 years old and has been used off label in younger children [3, 4]. The mechanisms of action of VNS are not completely understood. VNS is theorised to act by increasing the activity in the nucleus tractus solitarius and modulate a complex network of communication between the brainstem and cerebral structures [3, 4, 9]. Nucleus tractus solitarius projects to multiple brainstem centres, including the locus coeruleus, dorsal raphe nucleus, and parabrachial nucleus. Higher-order fibres project diffusely to cortical regions such as the thalamus, somatosensory cortex, anterior cingulate, and prefrontal cortex. Through these pathways, VNS leads to modulation of thalamocortical circuits and desynchronization of epileptiform activity. In addition, the delayed therapeutic effects that emerge over several weeks to years suggest network remodelling [7].

The largest multicentre, randomised trials of VNS outcomes in adults demonstrated the efficacy of high intensity stimulation (typical parameters: 1.5 mA output current, 30 Hz frequency, 500 ms pulse width, 30 s/5 min on/off time) compared to low intensity stimulation (1.25 mA, 1 Hz, 130 ms, 30 s/90 min on/off time) [10]. A randomized controlled trial (RCT) of 41 pediatric patients comparing high-output stimulation (output current 1.75 mA, pulse width 500 ms, 30 Hz frequency, 30 s/5 min on/off time) and low-output stimulation (0.25 mA, 100 ms, 1 Hz, 14 s/60 min on/off time) showed a greater than 50% reduction in seizure frequency in about 20% of patients, with no significant difference reported between groups [11]. A multicentre VNS registry which included participants with a diverse geographical and ethnic background found that most patients waited more than 5 years after epilepsy diagnosis for VNS implant and about two-thirds were responsive to therapy (> 50% reduction in seizure frequency) [12]. A large study by the Pediatric VNS group showed increasing seizure reduction with time after implant; a median seizure frequency reduction of 23% was observed after 3 months and reduction further increased to 31% at 6 months, 34% at 12 months, and 42% at 18 months [13]. Other potential benefits such as improvement of mood and cognition need further study [3, 4, 14]. Advances such as addition of auto-stimulation capability to detect and respond to ictal tachycardia (used as a biomarker for seizures) by additional stimulation at that time may improve its efficacy [15]. Nevertheless, about half the patients fail to present satisfactory response to VNS and there is a need to identify biomarkers (e.g. age, duration of epilepsy, type of epilepsy) of treatment success [7]. Adverse effects associated with VNS implants include infection, hematoma, vocal cord paralysis, facial weakness, pain and sleep disturbance. Overall, VNS serves as viable palliative treatment for pediatric patients with DRE. Acute implantation of VNS has been trialled to treat refractory status epilepticus in a few patients, but its effectiveness is not clear at present [5].

The cost of Vagus Nerve Stimulation Surgery in India varies depending on several factors, including the hospital, the surgeons fee, the complexity of the procedure, and the patient’s medical condition. Vagus Nerve Stimulation cost in Hyderabad ranges from Rs. 1,50,000 to Rs. 3,80,000 (https://www.yashodahospitals.com/). The cost may be slightly higher in private hospitals compared to government-run hospitals.

## Deep Brain Stimulation

Deep brain stimulation (DBS) was approved for use in adults with intractable epilepsy after the SANTE (Stimulation of the Anterior Nucleus of the Thalamus for Epilepsy) multicentre RCT involving 110 adult patients with focal refractory epilepsy [16]. Follow-up of these patients revealed ongoing progressive seizure improvement and a median percent seizure reduction of 69% after 5 years [17]. Stimulation of the hippocampus for treatment of refractory temporal lobe epilepsy and the centromedian nucleus of the thalamus (CNT) for Lennox-Gastaut syndrome has been found to be effective, but there is less evidence for benefits of stimulating other targets such as the subthalamic nucleus, the globus pallidus, the cerebellum, the caudate nucleus and the seizure onset zone [3, 4, 18]. Overall, the most frequently studied DBS targets with robust clinical trials demonstrating benefits are the anterior nucleus of the thalamus (ANT), hippocampus and CNT. Benefits of other targets remain inconclusive due to lack of evidence (open label trials or contradictory small-scale controlled trials). The antiepileptic effects of DBS vary extensively among individuals and potential factors which may predict efficacy include electrode location, type of epilepsy, normal MRI brain scan and stimulus parameters used. For example, ANT-DBS has been shown to be more effective when using high-frequency stimulation between 90 and 200 Hz with 90–160 μs pulse width and 1.5–10 V intensity.

The experience and information on DBS for children with DRE is limited, emphasizing the need for high quality RCTs in this age group. ANT- and CNT-DBS was found to be effective in children with focal and generalized epilepsy, respectively [3, 7, 19]. Add-on DBS has been found to be of benefit in children who have not responded to VNS therapy after a suitable interval [20].

Most DBS safety data come from movement disorders such as Parkinson’s Disease, while such data from epilepsy are less well documented. Main surgical complications are hemorrhage, infarction, and infection, while equipment related complications include lead fracture, malposition or migration. Other reported adverse effects include postoperative headache and confusion, paraesthesia, dysarthria, and cognitive disturbance. Overall rates of major complications varied from 1–4%. Long term follow-up of epilepsy patients did not reveal significant deterioration in cognition or depression scores [18].

Our current understanding of the mechanisms of action of DBS is limited; it is thought to disrupt networks involved in seizure propagation [3, 4, 8]. ANT has extensive connections to the hippocampal formation, cingulate cortices and inferior parietal lobule and ANT-DBS is thought to modulate limbic and thalamocortical networks. CNT has widespread connections with the striatum, other thalamic nuclei, reticular formation and sensorimotor cortex and modulation of cortico-thalamocortical circuits by CNT-DBS is a proposed mechanism of action.

The cost of Deep Brain Stimulation Surgery in India varies depending on several factors, including the hospital, the surgeons fee, the complexity of the procedure, and the patient’s medical condition. The average cost of Deep Brain Stimulation in India is approximately Rs. 8,00,000 to 15,00,000 (https://www.yashodahospitals.com/).

## Responsive Neurostimulation

A responsive neurostimulation (RNS) device is a closed loop device that activates solely during seizures [21]. RNS is particularly well-suited to treat seizures arising within eloquent cortex and therefore not candidates for surgical resections. In potential candidates for RNS, up to two seizure foci can be identified using intracranial EEG for subsequent simultaneous recording and stimulation by the implanted device. A predefined seizure-detection algorithm is used to detect ictal activity and deliver stimulation to the putative seizure onset zone to abort seizure activity. Seizure foci are identified using intracranial EEG for subsequent simultaneous recording and stimulation by the implanted device. The primary mechanism of action is through desynchronization of epileptiform activity at its source by membrane hyperpolarization and/or axonal conduction blockade. RNS may also function by suppressing cortical synchronization, even in regions distal from the area of stimulation by altering the plasticity of seizure networks [4, 9]. It was approved for treatment in adults after evidence of benefit from a RCT [22]. There is limited data in children. In an open label study Panov, et al. showed > 50% reduction in seizures in nearly 70% of children followed up over 1 year [23]. RNS has also been trialled targeting the CNT in pediatric and adult populations with LGS and generalized seizures, showing reduced seizure frequency, duration, and severity, improved post-ictal state and improved quality of life at long-term follow-up [24]. RNS reduces seizure frequency across a spectrum of pediatric epilepsy syndromes, irrespective of the seizures’ focal, multifocal or generalized origins [25]. Further studies are needed to inform clinical practise and there are clinical trials in progress including an FDA-approved trial of RNS in children with DRE (RNS System Responsive Thalamic Stimulation for Primary Generalized Seizures – NAUTILUS). Reported adverse events with RNS in children are rare and include haemorrhage, infection and device malfunction.

## Chronic Subthreshold Cortical Stimulation

Chronic subthreshold cortical stimulation (CSCS) is a form of neurostimulation consisting of continuous or cyclic, open-loop, subthreshold electrical stimulation of a well-defined seizure onset zone. In the study by Lundstrom, et al. [26] intracranial electroencephalography monitoring was performed with surgically implanted subdural grid and depth electrodes to accurately estimate the seizure focus followed by a therapeutic trial of continuous cortical stimulation (biphasic; frequency, 2–100 Hz; pulse width, 90–450 ms; amplitude, 1–6 V in voltage mode) via adjacent strip and occasional depth electrodes in the region of seizure onset. Permanent stimulation hardware (16-contact Medtronic Prime Advanced Neurostimulator with Medtronic 6-mm^2^ platinum-iridium 2 × 8 surgical leads or Medtronic DBS electrodes; model 3387, 3389, or 3391) was implanted when intracranial electroencephalography electrodes were explanted. Several studies have shown reduction in seizure frequency of more than 80% and improvement in quality-of-life following CSCS [26, 27]. Clinical outcome data in children is limited [28] and more studies are needed. In children stimulus parameters need to be individualized; in a case series of 3 children high-frequency stimulation improved seizures in two patients but worsened them in another; conversely, low frequency stimulation halted seizure activity in one and exacerbated it in another [28]. Mechanisms of action are currently not well understood. It has been proposed that subthreshold high-frequency stimulation can suppress intrinsic firing within cell soma. On the other hand, low-frequency stimulation may elicit long-term depression. Variability in responses may be due to patient or disease specific factors and may even relate to the specific orientation of the stimulation field with respect to the seizure focus [28]. Early tolerability data are promising, and it may be reasonable to expect a similar safety profile to other forms of intracranial stimulation.

## Non-Invasive Brain Stimulation

The main techniques for non-invasive brain stimulation are Transcranial Magnetic Stimulation (TMS), Transcranial Electrical Stimulation, Transcranial Photobiomodulation and Transcranial Ultrasound Stimulation [29]. Of these, TMS and transcranial electrical stimulation are the most common techniques being trialled for treatment of DRE [6, 30]. While a single session of non-invasive brain stimulation induces rather short-term effects (minutes up to hours), the application of non-invasive brain stimulation over multiple sessions (for several days/weeks) generates significantly longer lasting biological outcomes (in the order of weeks or a few months). The evidence of clinical changes that persist well beyond the time of stimulation is the basis for therapeutic and rehabilitative interventions [31].

## Transcranial Magnetic Stimulation (TMS)

Transcranial magnetic stimulation (TMS) induces a focal electric current in the cerebral cortex by a fluctuating extracranial magnetic field generated, most commonly, by a handheld electromagnet [6, 30]. Single or paired-pulse stimulation is used to measure cortical excitability while repetitive TMS (rTMS) can be used to increase or decrease excitability of the stimulated brain area depending on the frequency, pattern and duration of stimulation [6]. Several lines of evidence support the hypothesis that rTMS affects the brain by mechanisms like Long-Term-Potentiation and Long-Term-Depression [31]. Cortical inhibition induced by low frequency rTMS is most often investigated as treatment for patients with focal epilepsy [6].

Therapeutic rTMS has been used to treat ongoing seizures in the ictal state as well as improve seizure burden by one or more sessions of rTMS in the inter-ictal state. There are several case reports and case series describing the benefits of treating patients with ongoing seizures (refractory status epilepticus, epilepsia partialis continua). In these studies, seizure cessation was achieved in more than half the patients for varying durations (from 20–30 min to 2 months) and using varied stimulation parameters [30]. There was no identifiable relationship between stimulation frequency paradigms used and the degree of seizure reduction. Results are encouraging but need to be reinforced by RCTs to get a better understanding of treatment protocols for refractory status epilepticus and epilepsia partialis continua.

Controlled and open label rTMS trials have reported seizure reduction and decreased interictal epileptiform discharges (IEDs) in both adults and children with focal epilepsy. However, data in children is limited to case reports or inclusion of a few children in studies of mainly adult participants [3, 30–33]. About half the studies report a significant reduction in seizure frequency. Studies involving patients with generalized or multifocal epilepsy, focal epilepsy originating from deep foci, and coil placement over the vertex rather than over the seizure onset zone were less likely to report positive outcomes [30]. On average there is a 30% reduction of seizure frequency following rTMS with varying stimulus parameters and coil types [32]. Stimulation frequencies varied from 0.3–1 Hz, amplitude from 90–110% of resting motor threshold, and treatment was given in a single session, or biweekly for 4 weeks, or daily for 2 weeks, or 3 sessions a day for 2 weeks, or every day for 5 days, or twice a week for 3 months. Follow-up duration usually varied from 2–8 weeks. A recent meta-regression analysis suggests that rTMS has only a short-term effect on seizure frequency [33]. Overall, the benefits of rTMS in treating focal epilepsy in adults appear to be inconsistent and of short duration. Variability in patient characteristics, stimulation parameters, coils used, duration of treatment and follow-up, and heterogeneous etiology of epilepsy contribute to the mixed data. There are no controlled trials in children and further study is needed to identify children likely to benefit from rTMS and the most effective stimulation protocols.

TMS is usually well tolerated but rarely patients have been reported to have seizures during rTMS, more likely with high frequency rTMS. Other adverse effects of TMS and rTMS are usually mild and transient, and include headache, dizziness and discomfort during stimulation, and pain, nausea and sleep disturbance [6, 30]. There is less data in children, but adverse effects of TMS appears to be similar to adults.

## Transcranial Direct Current Stimulation

Transcranial Electrical Stimulation passes a low amplitude current (typically 1-2 mA) through active and return electrodes attached to the scalp [29]. The most common type of electrical stimulation is Transcranial Direct Current Stimulation (tDCS) although other forms of stimulation including Alternating Current Stimulation and Random Noise Stimulation have also been used. The effects of tDCS in the underlying cortex (excitatory or inhibitory) is dependent on the polarity of the active electrode; anodal is excitatory while cathodal is inhibitory. Stimulation with tDCS modulates neuronal excitability without reaching the threshold needed to trigger action potentials. Other mechanisms by which tDCS may induce long-term changes in the activity of neurons, connections, and networks include membrane oscillation, modulation of LTP (through activation of NMDA receptors), changes in neurotransmitter levels including GABA and Dopamine, protein synthesis and secretion of growth factors [31].

There have been many trials of cathodal tDCS for treatment of DRE in adults and children [3, 34, 35]. Most studies have reported some improvement in seizures after tDCS in adults and children with focal epilepsy [34]. Some have shown reduction in IEDs following tDCS [34, 35]. Positive results (a decrease in seizure frequency) were also reported in those with Lennox–Gastaut syndrome (LGS), Rasmussen’s encephalitis, epilepsia partialis continua, epileptic spasms, and Ohtahara syndrome [3, 34, 35]. A few studies have reported the failure of tDCS to improve seizure frequency. Follow-up has been relatively short, with most successful trials reporting improved seizure frequency over 1 month. Successful trials have used a wide range of stimulus parameters. One to fourteen once daily sessions were used, stimulus currents were from 1 to 2 mA, and the stimulus duration was 20–40 min for each session. The relationship between stimulus parameters and magnitude and duration of benefit are not clear. There have been fewer studies in children with varied improvement of seizures (from marginal to marked reductions in seizure frequency). Overall, the evidence suggests that cathodal tDCS may be potentially beneficial for treatment of DRE but additional RCTs with longer follow-up are needed to inform clinical practise.

Stimulation with tDCS appears to be well tolerated by adults and children and no serious adverse effects have been reported [34, 35]. Rare seizures have been reported in patients with DRE undergoing cathodal tDCS with no long-term worsening of seizures. Transient mild headache and local itchiness/irritation is often reported by patients during tDCS.

## Conclusion

Neurostimulation is a viable option for children with DRE who are not suitable for resective surgery. VNS is the most widely available technology and has been well studied in children. DBS, RNS and CSCS show encouraging results in adults but need further evaluation in children with well-designed RCTs. Similarly, further studies are needed for evaluating the efficacy of TMS and tDCS (and other NIBS techniques) in children with DRE and to determine optimal stimulation paradigms for long term improvement in seizure burden and quality of life. Neurostimulation has been tried in few cases for the treatment of refractory status epilepticus in adults but there is limited evidence for their use in children. Many of these techniques require high levels of expertise for the implantation and programming of the devices, which are available only in select clinical centres. There are no guidelines for choosing the best device for each patient and no reliable biomarkers to predict treatment response. Further studies are needed to provide clinical evidence for appropriate patient selection and develop personalised therapies.

### Key message

- About a third of children with epilepsy have drug resistant epilepsy (DRE, defined as failure to attain seizure freedom despite adequate trials of anti-seizure medications), emphasizing the need for alternative therapies.
- Surgical resection and dietary therapy are appropriate for a selected group of patients.
- Invasive and non-invasive devices are available and newer devices and techniques are being developed.
- The commonly available neurostimulation devices include vagus nerve stimulation (VNS), deep brain stimulation, responsive neurostimulation, chronic subthreshold cortical stimulation, transcranial magnetic stimulation and transcranial direct current stimulation.
- VNS is the best studies neurostimulation technique and has emerged as an important treatment option for children with DRE.
- Information for other devices is limited in children and they are not currently approved for use in children. Suitably designed controlled trials are needed for appropriate patient selection and reliable protocols.

## Data Availability

Not applicable.
